# Knowledge and practices toward COVID-19 among healthcare students: A cross-sectional study at the University of Zambia

**DOI:** 10.3389/fpubh.2022.1028312

**Published:** 2022-11-30

**Authors:** Steward Mudenda, Nelly Ngalande, Moses Mukosha, Christabel Nang'andu Hikaambo, Victor Daka, Scott Kaba Matafwali, Michelo Banda, Ruth Lindizyani Mfune, Godfrey Mayoka, Bwalya Angel Witika

**Affiliations:** ^1^Department of Pharmacy, School of Health Sciences, University of Zambia, Lusaka, Zambia; ^2^Department of Public Health, Michael Chilufya Sata School of Medicine, Copperbelt University, Ndola, Zambia; ^3^Clinical Research Department, Faculty of Infectious and Tropical Diseases, London School of Hygiene and Tropical Medicine, London, United Kingdom; ^4^Department of Pharmacology and Pharmacognosy, School of Pharmacy, Jomo Kenyatta University of Agriculture and Technology, Nairobi, Kenya; ^5^Department of Pharmaceutical Sciences, School of Pharmacy, Sefako Makgatho Health Sciences University, Pretoria, South Africa

**Keywords:** COVID-19, healthcare students, knowledge, practices, Zambia

## Abstract

**Background:**

The COVID-19 pandemic led to the disruption of physical classes for university students globally, as large gatherings fuelled the transmission of the virus. In the efforts to mitigate its transmission and return to normality, prevention measures, including vaccination, have been encouraged. Therefore, it is critical to understand the knowledge and practices of students regarding COVID-19. This study assessed the knowledge and practices toward COVID-19 among healthcare students at the University of Zambia.

**Materials and methods:**

This questionnaire-based cross-sectional study was carried out from August 2021 to October 2021 among 478 healthcare students (pharmacy, physiotherapy, nursing, biomedical, medicine, and radiography). We used a previously validated questionnaire to measure knowledge and practice. The predictors of knowledge and practices were assessed using logistic regression with robust estimation of standard errors. Statistical analysis was conducted using Stata/BE version 17.0.

**Results:**

Of the 478 respondents, 243 (50.8%) were females. A larger proportion, 175 (36.6%) were in Pharmacy training, and 156 (32.6%) were in their fifth year of study. The overall mean knowledge score of the participants was 87.9 (SD = 16.1), being higher at 89.6 (SD = 14.3) among medical students and the lowest at 86.7 (SD = 17.1) among Pharmacy students, although this was statistically non-significant (*p* = 0.488). The overall mean practice score was 60.0 (SD = 24.7), being significantly higher at 63.5 (23.4) among nursing, physiotherapy and environmental students compared to other students (*p* = 0.048). In multivariable analysis, the participant training program was non-significantly associated with knowledge and practice toward COVID-19. However, increased age (AOR = 1.09, 95% CI: 1.01–1.117) and residing in urban areas (AOR = 1.79, 95% CI: 1.07–3.01) than in rural areas were associated with higher odds of good practice toward COVID-19.

**Conclusion:**

The healthcare students generally showed good knowledge levels and poor practices toward COVID-19. Further, there was no evidence of a difference in knowledge of COVID-19 among healthcare students. These findings suggest the need for implementation strategies to be centered on improving the practices of students toward COVID-19.

## Introduction

Pandemics like the coronavirus disease 2019 (COVID-19) can potentially disrupt university education activities (1–3). This may eventually affect students' academic performance and social life (4–6). In addition, evidence has suggested that COVID-19 affected many people's mental health, including university students (7–10). This could be attributed to increased transmission and spread of the disease among students (11–14). Therefore, to curb disease transmission, face-to-face learning was suspended in many learning institutions globally (15–19).

The knowledge of individuals concerning COVID-19, its transmission, spread, and clinical features is significant in developing prevention strategies (20–22). Critical aspects of COVID-19-related knowledge required to illicit good practices have been postulated, including spread, symptoms, transmission, protective measures and vaccines (23). As future health care service providers and disease prevention specialists, students of health-related disciplines are expected to demonstrate appreciable knowledge in COVID-19 etiology, transmission, treatment, prevention and control (20). Variable findings have been reported, including high knowledge among students in India (24, 25) and Vietnam (26). Conversely, low knowledge levels were observed in Poland and China (27, 28). A study in sub-Saharan Africa reported good knowledge among students in selected institutions (29).

Evidence has demonstrated that individuals who adhere to preventive measures such as wearing face masks tend to have lower risks of contracting COVID-19 (30). In addition, practicing adequate hand hygiene has also been reported to reduce the transmission of the virus (31–33). Most people frequently touch their eyes, nose, and mouth when such acts may cause much harm. The practice of handwashing with running water and using alcohol-based handsanitisers can significantly reduce microbial contamination (32, 34). Moreover, studies have indicated that social distancing, avoiding crowded places, wearing masks, and hand hygiene reduce the probability of contracting COVID-19 and other respiratory diseases (35, 36). Thus, good practices toward COVID-19 prevention measures may help reduce the transmission of the virus and the spread of the disease (21, 22, 37, 38).

A milestone in the fight against COVID-19 has been developing, deploying and administering vaccines (39–42). Vaccines are critical in promoting the immune system to fight against infections (39). However, due to their accelerated development, there have been inconsistencies in the acceptance of the vaccine across populations (43–45). For instance, among the general population, vaccine acceptance was 83% in Denmark (46), 64.5% in Malaysia (47), 63.4% in Lebanon (48), 47% in France and Hungary (46), and 33.4% in Zambia (49). Similarly, inconsistencies in vaccine acceptance have been reported among healthcare workers (HCWs) including94.9% in Singapore (50), 89.2% in the United Arab Emirates (51), 74.5% in Ethiopia (52), 63.8% in Sudan (53), and 45.6% in Egypt (54). Acceptance of COVID-19 vaccine among students was 87.4% in China (55), 55.8% in Sudan (56), 54% in the United States, 27.1% in Ethiopia (57), and 24.5% in Zambia (58). These variations in vaccine acceptance have been due to concerns regarding the safety and effectiveness of vaccines (44, 59–61). Vaccine beliefs, myths and misinformation have also contributed to increased vaccine hesitancy (62, 63). Alongside this, vaccines' high cost and availability reduce their overall uptake (44). Therefore, targeted interventions must tackle vaccine hesitancy and improve acceptance and uptake across all populations (61–63).

In Zambia, COVID-19 led to increased morbidity and mortality with some deaths being reported as brought in dead (64–66). Alongside this, there has been low adherence to the COVID-19 prevention measures which could promote spread of the disease (20, 67). Additionally, many factors have been reported to affect the adherence to the COVID-19 prevention measure viz a viz limited information on COVID-19, travel patterns and social movements, negative attitudes toward COVID-19 guidelines, structural and socioeconomic factors (67). Therefore, addressing these factors is critical in reducing the spread of the disease.

The fight against COVID-19 requires a collaborative approach among all healthcare providers, including healthcare students, to provide optimum patient care (26). Furthermore, healthcare students are the future healthcare workers and will be responsible for providing disease prevention strategies to the public. Consequently, it is crucial to determine health-related students' level of knowledge about COVID-19 and associated prevention practices. Unfortunately, in many countries, including Zambia, there is a dearth of information regarding the knowledge and practices of university students concerning COVID-19. As such, this study assessed the knowledge and attitudes of healthcare students regarding COVID-19 at the University of Zambia in Lusaka, Zambia.

## Materials and methods

### Study design, setting and population

This cross-sectional study was conducted among healthcare students (biomedical sciences, medicine, nursing, pharmacy, physiotherapy, and radiography) from August 2021 to October 2021. The students were enrolled at the University of Zambia, Ridgeway medical campus in Lusaka. As the leading university in training healthcare professionals in Zambia; it was a good starting point to understand the knowledge and practices of students regarding COVID-19. To be part of the study, a student had to be enrolled in human healthcare programs at the University of Zambia and willing to respond to the questionnaire after giving consent.

### Sample size and sampling technique

The sample size was estimated using Cochrane's formula;


$$\text{n}=\frac{{\text{Z}}^{2}\text{p}\times \left(1-\text{p}\;\right)}{{\text{d}}^{2}}.$$

With no previous study done in this setting based on the literature search, a conservative expected proportion of 50%, 95% confidence level, 5% margin of error, 10% non-response or incomplete response, and desired design effect of 1.2 was used to determine the sample size. A minimum of 423 sample size was determined to achieve a minimum power of 80% to detect the difference in knowledge by the program of study. The sampling procedure had three steps. Firstly, we grouped the students into blocks based on their program of study (biomedical sciences, medicine, nursing, pharmacy, physiotherapy, and radiography). Secondly, we stratified the students according to their year of study. All potential participants were identified using the class registered for all registered students. Finally, a simple random sampling technique (using computer-generated random numbers without replacement) was used to select a random sample of students from each program of study.

### Data collection tool

Data collection was conducted using previously validated questionnaire from a similar study (68). The questionnaire was reviewed by two experts from the University of Zambia. The resultant questionnaire had three sections comprising seven questions on socio-demographics of participants, six questions on knowledge and four questions on practices toward COVID-19. Each correct knowledge question was assigned a score of one and a wrong response was assigned a zero. The questions on practice were assigned a score of one for good practices, otherwise, a zero was assigned. A Cronbach's alpha score of >0.7 was acceptable and used to determine the internal consistency of the questions. The self-administered questionnaire was piloted using 30 undergraduate healthcare students, but the pilot study findings were not part of the analyzed data in the main survey. The piloting of the data collection tool revealed that each participant would take between 10 and 20 min to respond to the questions. Data collection was conducted by three data collectors trained in the data collection process. To increase the chances of meeting the desired sample size and fears of non-response due to the COVID-19 spread, we distributed 600 questionnaires to the potential participants.

### Study measures

The main outcome measures were knowledge and practice measured on a binary scale (coded as yes = 1, no = 0). For each scale (knowledge and attitude scales) the item scores were summed to create a percentage score. The continuous scores for knowledge and practice were categorized based on Bloom's cut-off value (60% or less as poor knowledge and practice, >60% as good knowledge and practice). The primary predictor was the student's training program (pharmacy, medicine, biomedical sciences, nursing, environmental health, radiography). Other variables measured were age (years), sex (male, female), residence (urban, rural), marital status (married, unmarried) year of study, and religion.

### Statistical analysis

All analyses took into account the clustering of students within programs of study through the robust estimation of standard errors, which also accounted for the stratification by year of study. We used both descriptive and analytical statistical methods. The Q-Q plots were used to assess the normality of continuous data. The Analysis of Variance (ANOVA) test was used to evaluate the differences in the overall scores among the healthcare students. To assess pairwise comparison, ANOVA was followed by the Bonferroni *post-hoc* test where appropriate.

Separate logistic regression models with robust estimation of standard errors were fitted with knowledge and practice as outcome variables. The adjustment variables were chosen based on *p*-values from the univariable logistic regression models with knowledge and practice as outcomes, respectively, using a significance level of 20%. The main estimates were the training program's unadjusted odds ratios (UOR) and adjusted odds ratios (AOR). While adjusting for potential confounders, adjusted odds ratios and 95% confidence intervals were estimated to evaluate the type of training program with a report of good knowledge and practice toward COVID-19. Interactions between the training program and significant modifying variables were assessed, and none reached any statistical significance. We used Stata/BE version 17.0 (Stata Corp., College Station, Texas, USA) for analysis, and significance level was set at 5%.

### Ethical considerations

This study was approved by the University of Zambia Health Sciences Research Ethics Committee (UNZAHSREC) with protocol ID of 202112030049. Participation was voluntarily and confidentiality was observed.

## Results

### Socio-demographic characteristics of the study participants

We enrolled 478 respondents with a median age of 24 years (IQR, 23–26), of whom 243(50.8%) were females. Approximately two-in-five 175(36.6%) of the respondents were in Pharmacy training and 156(32.6%) were in the fifth year of study. Nearly all 466(97.5%) were of Christian faith, and 430(90.0%) were not married. Furthermore, the majority, 405(84.7%), resided in the urban parts of Zambia. There was no evidence suggesting that knowledge (*p* = 0.919) and practice (0.247) toward COVID-19 differed among the students. However, there was a statistically significant difference in median age, residential area and marital status between those respondents who reported a good attitude toward COVID-19 and those who did not (Table 1).

**Table 1:** Socio-demographic characteristics of study participants, *N* = 478.

**Characteristic**	**Total population *n* (%)**	**Knowledge**		***P*-value**	**Practice**		***P*-value**
**(l0ptr0pt)3-4 (l0ptr0pt)6-7**		**Poor *n* = 25**	**Good *n* = 543**		**Poor *n* = 215**	**Good *n* = 263**	
		**(%)**	**(%)**		**(%)**	**(%)**	
**Program**							
Other^#^	141 (29.5)	7 (28.0)	134 (29.6)	0.919^b^	60 (27.9)	81 (30.8)	0.247^a^
Biomedical	85 (17.8)	4 (16.0)	81 (17.9)		36 (16.7)	49 (18.6)	
Medicine	77 (16.1)	3 (12.0)	74 (16.3)		30 (14.0)	47 (17.9)	
Pharmacy	175 (36.6)	11 (44.0)	164 (36.2)		89 (41.4)	86 (32.7)
Age (years) median (IQR)	24 (23–26)	24 (23–25)	24 (23–26)	0.939^c^	24 (22–25)	24 (23–27)	0.006^c^
**Sex**							
Female	243 (50.8)	11 (44.0)	232 (51.2)	0.482^a^	104 (48.4)	139 (52.9)	0.330^a^
Male	235 (49.2)	14 (56.0)	221 (48.8)		111 (51.6)	124 (47.2)	
**Year of study**							
Second	59 (12.3)	3 (12.0)	56 (12.4)	0.400^b^	26 (12.1)	33 (12.6)	0.229^a^
Third	96 (20.1)	2 (8.0)	94 (20.8)		48 (22.3)	48 (18.3)	
Fourth	131 (27.4)	7 (28.0)	124 (27.4)		62 (28.8)	69 (26.2)	
Fifth	156 (32.6)	12 (48.0)	144 (31.8)		69 (32.1)	87 (33.1)	
Above fifth	36 (7.5)	1 (4.0)	35 (7.7)		10 (4.7)	26 (9.9)	
**Marital status**							
Unmarried	430 (90.0)	22 (88.0)	408 (90.1)	0.730^b^	200 (93.0)	230 (87.5)	0.044^a^
Married	48 (10.0)	3 (12.0)	45 (9.9)		15 (7.0)	33 (12.6)	
**Residence**							
Rural	73 (15.3)	7 (28.0)	66 (14.6)	0.084^b^	42 (19.5)	31 (11.8)	0.019^a^
Urban	405 (84.7)	18 (72.0)	387 (85.4)		173 (80.5)	232 (88.2)	
**Religion**							
Other^*^	12 (2.5)	1 (4.0)	11 (2.4)	0.479^b^	7 (3.3)	5 (1.9)	0.346^b^
Christian	466 (97.5)	24 (96.0)	442 (97.6)		208 (96.7)	258 (263)	

a Pearson Chi-square test,

b Fishers exact test,

c Wilcoxon rank sum test,

* minor religions in Zambia (Hindu, Islam, Buddhist, etc.),

# health sciences students (nursing, environmental health, radiography).

### Knowledge of COVID-19 among healthcare students

The knowledge statements and percentage of correct responses from the participating healthcare students are shown in Table 2. Overall, the mean knowledge score of the participants was 87.9 (SD = 16.1), the highest score 89.6 (SD = 14.3) arising from medical students and the lowest 86.7 (SD = 17.1) from Pharmacy students, although this was statistically non-significant (*p* = 0.488). The most correctly answered question among the participants was on the clinical symptoms of COVID-19 infection (96%), and the least was on whether eating or contacting wild animals would result in infection with COVID-19 (77.8%). When different questions on knowledge of COVID-19 were compared among the participating healthcare students, a significant difference was found with a question on the clinical symptoms of COVID-19 (*p* = 0.009).

**Table 2:** Percentage of correct responses to knowledge statements.

**Knowledge statements**	**Total *n* = 478 (%)**	**Biomedical *n* = 85 (%)**	**Medicine *n* = 77(%)**	**Pharmacy *n* = 175(%)**	Other^*^n = 141(%)	***P*-value**
COVID-19 spreads *via* respiratory droplets from infected individuals	454 (95.0)	79 (92.9)	76 (98.7)	162 (92.6)	137 (97.2)	0.087^a^
The clinical symptoms of COVID-19 include headache, sore throat, fever, fatigue, dry cough, and myalgia	459 (96.0)	84 (98.8)	69 (89.6)	172 (98.3)	134 (95.0)	0.009^a^
Currently, there is no effective cure for COVID-2019, but early symptomatic and supportive treatment can help most patients recover from infection	418 (87.5)	76 (89.4)	70 (90.9)	148 (84.6)	124 (87.9)	0.477^b^
Not all persons with COVID-2019 will develop severe cases. Though the elderly and those with chronic illnesses are more likely to be in severe cases	375 (78.5)	64 (75.3)	63 (81.8)	138 (78.9)	110 (78.0)	0.789^b^
Wild animals are sources of COVID-19 infections	372 (77.8)	63 (74.1)	62 (80.5)	129 (73.7)	118 (83.7)	0.137^b^
Wearing face masks can prevent COVID-19	442 (92.5)	79 (92.9)	74 (96.1)	161 (92.0)	128 (90.8)	0.548^b^
Overall mean (SD)	87.9 (16.1)	87.3 (15.8)	89.6 (14.3)	86.7 (17.1)	88.8 (16.0)	0.488^c^

a Fishers exact test,

b Pearson Chi-square test,

c One-way analysis of variance (ANOVA),

* health sciences students (nursing, environmental health, radiography), all values are mean and standard deviations.

### Practice toward COVID-19 among healthcare students

The practice statements and percentage of correct responses from the participating healthcare students are shown in Table 3. Overall, the mean practice score of the participants was 60.0 (SD = 24.7), being significantly higher at 63.5 (SD = 23.4) among other students (nursing, physiotherapy and environmental health students) compared to biomedical, medicine and pharmacy students (*p* = 0.048). Most 409 (85.6%) students reported wearing facial masks often when in public. On the other hand, the majority, 303 (63.4%) reported that they did not avoid visiting crowded places. When study programs were compared, a statistically significant difference was observed across all practice questions. When different questions on practice toward COVID-19 were compared among the participating healthcare students, a significant difference was found with all the questions.

**Table 3:** Percentage of correct responses to practice statements.

**Practice statements**	**Total *n* = 478 (%)**	**Biomedical *n* = 85 (%)**	**Medicine *n* = 77(%)**	**Pharmacy *n* = 175(%)**	**Other^a^ *n* = 141(%)**	***P*-value**
I often wear facial masks when in public	409 (85.6)	69 (81.2)	69 (89.6)	141 (80.6)	130 (92.2)	0.012
I practice hand washing and hand sanitizing regularly	352 (73.6)	61 (71.8)	62 (80.5)	116 (66.3)	113 (80.1)	0.019
I avoid visiting crowded places	303 (63.4)	64 (75.3)	42 (54.6)	112 (64.0)	85 (60.3)	0.038
I am willing to receive the COVID-19 vaccine to protect myself from the disease	394 (82.4)	75 (88.2)	57 (74.0)	151 (86.3)	111 (78.7)	0.031
Overall mean (SD)	60.0 (24.7)	60.0 (24.8)	62.7 (23.9)	56.1 (25.9)	63.5 (23.4)	0.048

All values are mean and Standard Deviation (SD) and p-value from Pearson Chi-square tests; otherwise, One-way Analysis of Variance (ANOVA) was used,

a health sciences students (nursing, environmental health, radiography).

### Factors associated with knowledge and practice toward COVID-19

The univariable and multivariable results from a logistic regression analysis are depicted in Table 4. The univariable analysis showed no association between participants' training program, practice, and knowledge of COVID-19. Multivariable analysis was further used to evaluate participants' training program while adjusting for potential confounders. In multivariable analysis, the participant training program remained non-significantly associated with knowledge and practice toward COVID-19. However, age and residence (Urban compared to Rural) were positively associated with practice toward COVID-19. A unit increase in the participant's age was associated with higher odds of good practice toward COVID-19 (AOR = 1.09, 95% CI: 1.01–1.117). In addition, participants who resided in urban areas were more likely to have good practices toward COVID-19 (AOR = 1.79, 95% CI: 1.07–3.01) than those who resided in rural areas.

**Table 4:** Simple and multiple logistic regression models.

**Characteristic**	**Knowledge**		**Practice**	
	**UOR (95% CI)**	**AOR (95% CI)**	**UOR (95% CI)**	**AOR (95% CI)**
**Program**				
Other^#^	1.00	1.00	1.00	1.00
Biomedical	1.06 (0.300–3.73)	1.05 (0.30–3.73)	1.16 (0.66–2.05)	1.15 (0.66–2.01)
Medicine	1.29 (0.32–5.13)	1.22 (0.31–4.89)	0.72 (0.46–1.12)^b^	0.90 (0.44–1.83)
Pharmacy	0.78 (0.29–2.06)	0.79 (0.30–2.10)	(0.58–1.74)	0.75 (0.47–1.19)
**Age (years**) median (IQR)	1.01 (0.90–1.14)	–	1.09 (1.03–1.15)^b^	1.09 (1.01–1.17)^a^
**Sex**				
Female	1.00	–	1.00	–
Male	0.75 (0.33–1.68)		0.84 (0.58–1.20)	
**Year of study**				
Second	1.00	–	1.00	1.00
Third	2.52 (0.41–15.53)		0.79 (0.41–1.51)	0.59 (0.29–1.18)
Fourth	0.95 (0.24–3.81)		0.88 (0.47–1.63)	0.67 (0.34–1.34)
Fifth	0.64 (0.17–2.36)		0.99 (0.54–1.82)^b^	0.71 (0.35–1.42)
Above fifth	1.88 (0.19–18.74)	2.05 (0.84–5.00)	1.35 (0.42–4.32)
**Marital status**				
Unmarried	1.00	–	1.00	1.00
Married	0.81 (0.23–2.81)		1.91 (1.01–3.62)^b^	1.13 (0.51–2.53)
**Residence**				
Rural	1.00	1.00	1.00	1.00
Urban	2.28 (0.92)^b^	2.23 (0.89–5.56)^&^	1.82 (1.10–3.01)^b^	1.79 (1.07–3.01)^a^
**Religion**				
Other^*^	1.00	–	1.00	–
Christian	1.67 (0.21–13.51)		1.74 (0.54–5.55)	

UOR, unadjusted odds ratio; AOR, Adjusted odds ratio,

& borderline evidence,

* minor religions in Zambia (Hindu, Islam, Buddhist, etc.),

# health sciences students (nursing, environmental health, radiography),

a significant at p < 0.05,

b significant at p < 0.2, in the model for knowledge, program was retained as a priori variable.

## Discussion

We believe this is the first comprehensive study on knowledge and practices among healthcare students in Zambia to provide baseline data regarding COVID-19 in tertiary learning institutions. In addition, provide key areas to inform future quality improvement efforts and capacity development of COVID-19 response and preventive measures in Zambia. The latter is important as there have been concerns with knowledge and practices regarding COVID-19 among healthcare students in Zambia (20). In the present, overall, we found an average knowledge and attitude score of 89.6 and 60% among healthcare students. Medical students were more knowledgeable about COVID-19 causes, spread, and prevention than other students, while nursing, physiotherapy, and environmental health students reported good practices toward COVID-19 prevention measures than other students. Even though the students' training program was not independently associated with knowledge and practice toward COVID-19, increased age and residing in urban areas (compared to rural) predicted higher odds of good practice toward COVID-19.

The overall healthcare students' knowledge of COVID-19 is consistent with the extant literature (69, 70). For instance, a study in Vietnam found that most students had good knowledge (86.6%) about COVID-19 and the prevention measures (26), similar to findings from Ethiopia (70). However, our findings suggest that the level of knowledge of COVID-19 was not independently associated with students' programs of study, which is contrary to findings from a study done in Poland where significant differences were observed between students of different training programs (27). Although no significant difference was reported regarding knowledge of COVID-19 across study programs in our study, medical students scored higher compared to other study programs. This is similar to what was found in Poland in which medical students had better knowledge of COVID-19 compared to other students from other programs (27). These findings could be attributed to the fact that medical students are exposed to clinical practice early and attend several hospital meetings. Nevertheless, the findings are encouraging as they indicate that future healthcare workers have sufficient knowledge of COVID-19 which is key in developing preventive measures for this pandemic.

The participants in this study were knowledgeable about the spread of the disease, clinical features, treatment, predisposed individuals to severe disease, and wearing face masks. These findings corroborate reports from Iran in which students had good knowledge regarding COVID-19 transmission and spread, symptoms, and wearing face masks (71). This knowledge, however, should be enhanced by providing students with information regarding the proper use and different types of facemasks.

The current study highlighted the poor practices of students regarding COVID-19, similar to findings reported in studies conducted among university students (13, 69, 70, 72). For instance, a study in Indonesia reported an overall practice of 51.5% among university students (69), in line with findings from Ethiopia (70). Most participants reported wearing facemasks in public, which corroborate findings from other studies (69, 73). However, the current findings are higher than those reported by Kateule and others in an observational study where 24% of the participants wore masks in Lusaka district and 27% wore masks in Mansa district of Zambia (74). These differences could be attributed to differences in study designs and socio-demographic characteristics of study participants. Therefore, wearing face masks during outbreaks of respiratory infections should be promoted as a public health disease prevention and control strategy.

Overall, most participants in this study reported handwashing and sanitizing regularly. However, the percentage of compliance was lower than those reported in 10 countries in Africa through a multinational survey (75). While avoiding crowded places is a key COVID-19 intervention strategy, it was observed in our study that there was less inclination to avoid crowded places than what was reported in a similar study done in the Netherlands (76). The majority (82.5%) of participants in this study were willing to receive the COVID-19 vaccine, which was identical to observations reported in Lebanon (77), Bangladesh (78), China (55), and the Philippines (79). It is envisaged that increased vaccine acceptance may help increase vaccinations across the globe (80). An earlier study conducted immediately after vaccine deployment in Zambia reported a very low vaccine acceptance (24.5%) among pharmacy students (58). This could have been due to inadequate and negative information about the vaccines. Other studies have reported low vaccine acceptance among students with low vaccine acceptance attributed to misinformation, myths, and concerns about the adverse effects and effectiveness of vaccines (56, 57, 81–83).

Our study found that older participants observed COVID-19 prevention practices much better than younger participants, despite both groups displaying comparable levels of knowledge. These findings contrasts those from a study that was conducted among healthcare students in Vietnam whereby the pattern and extent of COVID-19 practices could not be distinguished along the age of the study participants (26). While it is unclear why age might have contributed to the discrepancy in the students' COVID-19-related safe practices, we posit that older age is generally associated with more responsible health behaviors. Moreover, those students who resided in urban areas tended to uphold safe hygiene and other preventive practices toward controlling possible COVID-19 transmission, compared to those who identified themselves as living in rural areas. Similar observations were reported in a survey among students in Japan where students who lived in the capital city scored highly compared to others, in following national and international measures recommended to mitigate against the spread of COVID-19 (68). It is conceivable that public health outreach programs that rely heavily on social media and other digital communication platforms are central to the observed differences. There is generally better penetration and access to information among the urban dwelling residents than rural residents. Also, in most cases, initial and severe cases of COVID-19 were reported in urban areas. This could have made the urban residents much more aware and conscious of the public health implications of the uncontrolled spread of the disease. Interestingly, rural-dwelling students in another African set-up in Ethiopia were twice as likely to comply with recommended public health measures to avert COVID-19 transmission, compared to their urban counterparts (84). Potential socioeconomic differences, the impact of the public health campaign strategies, and outreach that the two countries may have mounted, could be contributing factors to this observation.

Surprisingly, the reported good knowledge regarding COVID-19 across all students in our survey was at variance with the practices. Similar findings were reported from Ethiopia in which good knowledge did not translate into good practices toward COVID-19 (70). These findings may require multiple strategies to be implemented when disseminating COVID-19 information to college and university students. Conversely, a study in the Kingdom of Bhutan among college students found good knowledge that translated into good practices toward COVID-19 (85). Similarly, a recent study in Ethiopia reported good knowledge and good practices regarding COVID-19 (86). The good knowledge and practices reported in other studies could be due to the increased dissemination of educational information regarding COVID-19 by the governments and related stakeholders. Our findings and those from similar surveys may be used to develop strategies that limit disease spread.

This study had some limitations. First, it was conducted at one institution of higher learning, therefore, the findings may not be generalized to all the universities across the country. Secondly, the study focused on healthcare students, hence, the findings may not be generalized to non-healthcare students.

## Conclusion

The study found good knowledge of COVID-19 among university students. However, the overall poor practices are of much concern and require urgent attention from authorities. Despite the students having good knowledge, the poor practices in some infection prevention measures call for improved dissemination of COVID-19 information in universities and across the youth population. The findings from the study are hypothesis-generating and can guide implementation strategies aimed at improving the practices toward COVID-19.

## Data availability statement

The raw data supporting the conclusions of this article will be made available by the authors, without undue reservation.

## Author contributions

The study was conceptualized by SM and NN. Data collection was done by SM, NN, and CNH. Data analysis was done by SM and MM. Intellectual content of the manuscript was done by SM, MM, CNH, SKM, VD, MB, RLM, and BAW. Writing of the first draft was done by SM. All authors participated in the writing of the final manuscript, reviewed, and approved the final version of the manuscript.

## Conflict of interest

The authors declare that the research was conducted in the absence of any commercial or financial relationships that could be construed as a potential conflict of interest.

## Publisher's note

All claims expressed in this article are solely those of the authors and do not necessarily represent those of their affiliated organizations, or those of the publisher, the editors and the reviewers. Any product that may be evaluated in this article, or claim that may be made by its manufacturer, is not guaranteed or endorsed by the publisher.
