# Supplementary material for: Knowledge and practices toward COVID-19 among healthcare students: A cross-sectional study at the University of Zambia
Source: Front Public Health. 2022 Nov 30;10:1028312. doi: 10.3389/fpubh.2022.1028312 (PMC9748439; doi:10.3389/fpubh.2022.1028312)
Supplement: Supplementary file 1 [file Data_Sheet_1.PDF]

Knowledge and practices toward COVID-19 among healthcare students: A cross-sectional study at the University of Zambia

Section A: Demographic Characteristics of Participants

1. What is your gender? A. male [ ]   b. female [ ]
2. How old are you?
3. What program are you studying? a. pharmacy [ ] b. radiography [ ] c. Biomedical Sciences [ ] d. Environmental Health e. Physiotherapy [ ] f. Medicine
4. Which year of study are you in? a. 2nd yr. [ ] b. 3rd yr. [ ] c. 4th yr. [ ] d. 5th yr. [ ] e. above 5th year [ ]
5. What is your marital status? a. Married [ ] b. Unmarried [ ]
6. Which of these describes your current residence? a. Urban [ ] b. Rural [ ]
7. What is your religion? A. Christianity [ ] B. Islam [ ] C. Hinduism [ ] D. others specify

Section B: Knowledge of participants towards COVID-19

1. The COVID-19 spreads via respiratory droplets of infected individuals.
2. The clinical symptoms of COVID-19 include headache, sore throat, fever, fatigue, dry cough, and myalgia.
3. There currently is no effective cure for COVID-2019, but early symptomatic and supportive treatment can help most patients recover from infection.
4. Not all persons with COVID-2019 will develop severe cases, though the elderly and those with chronic illnesses are more likely to be in severe cases.
5. Eating or contacting wild animals would result in infection by the COVID-19 virus.
6. Ordinary residents can wear general medical masks to prevent infection by the COVID-19 virus.

Section C: Practices of participants towards COVID-19

1. I often wear face masks when in public
2. I practice hand washing and hand sanitising regularly
3. I avoid visiting overcrowded places
4. I am willing to receive the COVID-19 vaccine to protect myself from the disease

Thank you for your participation
